# Effects of Various Preextraction Treatments of *Crinum asiaticum* Leaf on Its Anti-Inflammatory Activity and Chemical Properties

**DOI:** 10.1155/2021/8850744

**Published:** 2021-01-28

**Authors:** Chonthicha Kongkwamcharoen, Arunporn Itharat, Weerachai Pipatrattanaseree, Buncha Ooraikul

**Affiliations:** ^1^Graduate School on Applied Thai Traditional Medicine Program, Faculty of Medicine, Thammasat University, Pathum Thani 12120, Thailand; ^2^Department of Applied Thai Traditional Medicine, Faculty of Medicine, Thammasat University, Pathum Thani 12120, Thailand; ^3^Center of Excellence on Applied Thai Traditional Medicine Research (CEATMR), Faculty of Medicine, Thammasat University, Pathum Thani 12120, Thailand; ^4^Regional Medical Science Center 12 Songkhla, Department of Medical Sciences, Songkhla 90100, Thailand; ^5^Department of Agricultural Food and Nutritional Science, Faculty of Agricultural Life and Environmental Sciences, University of Alberta, Edmonton, AB T6G 2P5, Canada

## Abstract

*Crinum asiaticum* Linn. has been used in Thai traditional medicine to relieve inflammatory symptoms and treat osteoarthritis. There have been reports on its potent anti-inflammatory property but nothing on the effects of different pretreatments on its chemical properties and anti-inflammatory activity. Pretreatment of herbal raw materials is an important step which affects the overall quality of Thai traditional medicine. The objectives of this study were to investigate different treatments of *C. asiaticum* leaves prior to ethanolic extraction and to compare the extracts for their anti-inflammatory activity and chemical properties. The treatments included hot air drying in an oven, microwave drying, traditional grilling on a charcoal stove before drying in an oven, and temperature shock in hot and cold water before hot air drying. The anti-inflammatory activity and chemical properties of the extracts were analyzed using the established methods. Results showed that 95% ethanolic extract of hot air oven-dried leaves had the highest anti-inflammatory activity and total phenolic and lycorine contents. We recommend hot air drying as a preextraction treatment for *C. asiaticum* leaves for its simplicity, best retention of the herbal quality, and suitability for scaling up to an industrial process.

## 1. Introduction

Inflammation is a primary physiologic response of tissue to pathogens, cell injury, and damaged tissues. Proinflammatory cytokines, inflammatory mediators such as nitric oxide (NO), prostaglandin E_2_ (PGE_2_), and tumor necrosis factor-alpha (TNF-*α*) produced during the inflammatory process cause damage of cell, tissue, and organ [1, 2]. Regarding the pathophysiology of inflammatory diseases, osteoarthritis (OA) is the most common chronic degenerative disease among elderly people. The inflammatory mediators such as nitric oxide, IL-1, TNF-*α*, and COX-2 contribute to cartilage and joint degeneration [3]. Nonsteroidal anti-inflammatory drugs (NSAIDs) are the common treatment for OA pain and inflammation. However, adverse effects have been observed in elderly patients [4].

*Crinum asiaticum* L. (Amaryllidaceae family) has long been used in Southeast Asian and Thai traditional medicine to relieve pain and as a treatment for inflammatory diseases. According to ethnopharmacology, Southeast Asian countries use *C. asiaticum* for treatments of wounds, swellings, pain injuries, and inflamed joints, and as an antidote for poisons or toxins [5–7]. In Thai traditional medicine, *C. asiaticum* leaf is a plant component in Phra-aung-kob-phra-sean remedy commonly used to treat injurious, inflamed joints [8], and ankle pain and for postpartum care [9].

Local practitioners grill *C. asiaticum* leaves on a charcoal stove until they are softened and hot before covering the localized pain areas. However, the effects of this traditional heating treatment on the inflammatory activity of the leaves have not been elucidated.

Previous researches have reported on the bioactivity of *C. asiaticum* leaves including antioxidation [10] and anti-inflammation by inhibiting nitric oxide production [11]. An *in vivo* study on leaf extract also showed a significant anti-inflammatory effect [12]. Among the phytochemical components in *C. asiaticum*, lycorine is the most common of *Crinum* alkaloids that exerts potent immunological, antitumor, and anti-inflammatory activities [13–15] and decreases autophagy in osteoclasts *in vivo* [16]. These studies have reported on the potent anti-inflammatory effect of *C. asiaticum* but so far no study has been done on the effects of different preextraction treatments on its chemical properties and anti-inflammatory activity. Our study was designed to fill this gap in knowledge.

Traditional Thai doctors usually have their own methods of preparation before they apply the herbs. These may involve heating, grilling, or pounding the fresh herb for topical applications, or boiling it in water or alcohol, if it is to be taken internally. These pretreatment techniques are applied for several reasons, for example, for long-term storage, quality control, increasing efficacy, and reducing the toxicity of the herbs [8].

Preextraction treatments are physical preparations of the herb which include cleaning, size reduction, and drying prior to solvent extraction. Ethanolic extraction with 95% ethanol is usually an extraction method of choice for most herbal materials for the purpose of scientific studies on their bioactive components and efficacy against target diseases. However, the quality of herbal extracts is usually influenced by the treatment of the herbs before extraction, especially the drying process. Drying temperature and time and how the drying process is applied to the fresh herb can have a profound effect on the chemical composition of the extracts and hence medicinal efficacy [17, 18].

In this research, we applied four methods of pretreatments to get *C. asiaticum* leaves ready for ethanolic extraction and to evaluate what effects each of them has on the chemical and anti-inflammation properties of the herb. The first method was grilling on a charcoal stove, a common practice in the Thai traditional medicine. The second method was hot air drying in an oven in which the temperature and time could be conveniently controlled in order to minimize damage to the leaves. The third method was microwave drying which employs direct electromagnetic heating to the material enabling ease of temperature control and faster drying to better retain herbal qualities. The fourth method was temperature shock in water which might help preserve color and some important substances in the leaves. We then compared the effects of the four preextraction treatments of *C. asiaticum* leaf on its anti-inflammatory activity, as determined by the inhibition of NO, PGE_2_, and TNF-*α* production, and its chemical properties such as total phenolic compound and lycorine content. From the results, we recommended the most suitable treatment that would best preserve the quality of *C. asiaticum* leaves for the development of an anti-inflammation product for pain relief in OA patients.

## 2. Material and Methods

### 2.1. Plant Materials and Preextraction Treatment Methods

**Figure 1:**
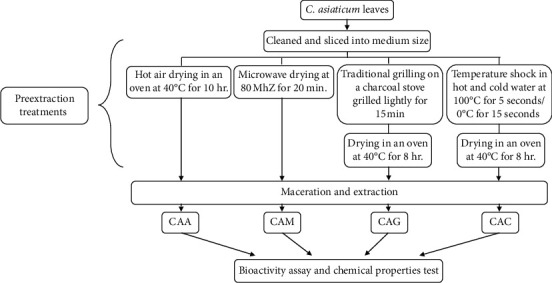
Concept diagram of the steps in the experiments; CAA means *C. asiaticum* extract of hot air drying in oven method, CAM means *C. asiaticum* extract of microwave drying method, CAG means *C. asiaticum* extract of traditional grilling on a charcoal stove method, and CAC means *C. asiaticum* extract of temperature shock method.

*C. asiaticum* leaves were collected from the Faculty of Medicine, Thammasat University, Pathum Thani, Thailand. The specimen voucher (SKP-008 03 01 01) was deposited at the herbarium of Southern Center of Thai Medicinal Plants, Faculty of Pharmaceutical Sciences, Prince of Songkla University, Thailand. The concept diagram of the steps in the experiments is shown in Figure 1.

Fresh leaves were cleaned with water and sliced into medium-size pieces before being subjected to the following four different preextraction treatments:

1. Hot air drying in an oven (CAA) with the oven set at 40°C for 10 hours.
2. Microwave drying (CAM) with a microwave power of 80°MhZ for 20 minutes.
3. Traditional grilling on a charcoal stove before drying in an oven (CAG): the leaves were placed on the grid of a charcoal stove and grilled lightly for 15 minutes before drying in a hot air oven at 40°C for 8 hours.
4. Temperature shock in hot and cold water before hot air drying (CAC): the leaves were dipped in a water bath at 100°C for 5 seconds and then immediately plunged into an ice-cold water bath (0°C) for 15 seconds before drying in a hot air oven at 40°C for 8 hours.

### 2.2. Maceration and Extraction Method

The dried leaves from each preextraction treatment (40 g) were macerated with 95% ethanol for three days and filtered through a Whatman No. 1 filter paper. The maceration was repeated twice and the extracts were combined. The crude extract was dried in a rotary evaporator.

### 2.3. Reagents and Chemicals

Standard lycorine (purity > 98%) was purchased from Chem Faces (Wuhan, China). Dimethyl sulfoxide (DMSO), phosphoric acid, triethylamine, and purified water were prepared by Milli Q® system from Millipore (Bedford, MA, USA). Acetonitrile HPLC grade was purchased from RCI Lab Scan (Bangkok, Thailand). Murine macrophage leukemia cell line (RAW 264.7: ATCC® TIB-71™) was purchased from American Type Culture Collection (ATCC®, VA, USA). Fetal bovine serum (FBS) was purchased from Gibco® (OK, USA). Dulbecco's Modified Eagle's Medium (DMEM), phosphate buffer saline (PBS), and penicillin-streptomycin (P/S) were purchased from Biochrom (MA, Germany). 3-(4, 5-Dimethyl-2-thiazolyl)-2, 5-diphenyl-2H-tetrazolium bromide (MTT) was purchased from Sigma (MO, USA).

### 2.4. Instruments

The HPLC system (Agilent® 1200; Agilent Technologies, USA) consisted of a solvent degasser (G1322A), an autosampler (G1329A), a quaternary solvent pump (G1311A), a photodiode array detector (G1315D), and a column oven (G1316A). The chromatographic data were processed by the Chemstation® software version B.04.01 SP1.

### 2.5. Anti-Inflammatory Activity by Inhibition of Nitric Oxide Production from RAW 264.7 Cells and Cytotoxicity by MTT Assay

Anti-inflammatory activity by inhibition of nitric oxide production in RAW264.7 cells was determined according to the method of Tewtrakul and Itharat, with slight modifications [19]. Briefly, RAW264.7 cells were cultured in DMEM medium with 10% fetal bovine serum (FBS), penicillin (100 units/mL), and streptomycin (100 *µ*g/mL) and incubated at 37°C under 5% CO_2_. The cells were seeded into 96-well plate and incubated for 24 hours. The cells were treated with LPS (lipopolysaccharide) at the final concentration of 5 ng/mL. A sample solution was added and incubated for 24 hours. The supernatant was transferred to a new 96-well plate and Griess reagent was added. The plate optical density (OD) was measured with a microplate reader at a wavelength of 570 nm. The viability of the treated cells was determined by MTT solution. The OD was measured at 570 nm. The %inhibition and %toxicity were calculated. The half inhibitory concentration (IC_50_) was determined by regression analysis using GraphPad Prism software (CA, USA).

### 2.6. Anti-Inflammatory Activity by Inhibition of TNF-*α* Production from RAW 264.7 Cells

Inhibitory effects on the TNF- *α* production from RAW 264.7 cells were evaluated by using Quantikine® mouse TNF-*α* ELISA test kit (Darmstadt, Germany) according to the method of Makchuchit et al. [20] with slight modifications. In brief, the cells were seeded in 96-well plates at a density of 1 × 10^5^ cells/well and incubated for 24 hours. After that, the medium was replaced with fresh medium containing 5 ng/mL of LPS and test samples at various concentrations and incubated for 24 hours. The supernatant was transferred into 96-well ELISA. The OD was measured at 450 nm. Inhibition percentage of TNF-*α* production was calculated and the Prism program used for calculating IC_50_ values.

### 2.7. Anti-Inflammatory Activity by Inhibition of Prostaglandins E2 (PGE2) from RAW 264.7 Cells Line

RAW264.7 macrophage cells were seeded in 96-well plates with 1 × 10^5^ cells/well and incubated for 18–20 hours. Afterward, the medium was replaced by a fresh medium containing 0.08 *μ*g/mL of LPS with various concentrations of sample and incubated for 24 hours. The supernatant was transferred into 96-well PGE_2_ ELISA plate (Cayman Chemical Company). The absorbance was measured at 420 nm. Inhibition percentage of PGE_2_ production was calculated and the Prism program used to calculate IC_50_ values.

### 2.8. Determination of Total Phenolic Content

The total phenolic content of the extracts was determined by the modified Folin–Ciocalteu method [21]. A 20 *μ*L aliquot of the extracts was mixed with 100 *μ*L of Folin–Ciocalteu's reagent and 80 *μ*L of sodium carbonate. The plate was mixed well and allowed to stand at room temperature to develop color for 30 minutes. The absorbance was measured at 765 nm. Total phenolic content was expressed as mg gallic acid equivalents (GAE)/g. It was calculated from a calibration curve of gallic acid standard solutions (ranging from 2.5 to 100 *μ*g/mL). All tests were performed in triplicate.

### 2.9. Determination of Lycorine Content by HPLC

#### 2.9.1. Preparation of Standard and Sample Solution

A stock standard solution of lycorine was prepared at a concentration of 0.5 mg/mL in DMSO. A calibration curve was constructed by using the serially diluted standard solution at the concentrations of 50, 100, 150, 200, 300, and 500 *µ*g/mL. For sample solutions, each *C. asiaticum* extract obtained from various preextraction treatments was dissolved in DMSO to produce a sample solution containing 10 mg/mL of the extract.

#### 2.9.2. Chromatographic Conditions

Chemical constituents of the crude extract were separated along a C18 reverse-phase column (Zorbax® C18, 4.6 × 250 mm, 5 microns). The mobile phase consisted of 0.1%v/v triethylamine in water adjusting pH to 3.0 with phosphoric acid (A) and acetonitrile (B). Gradient elution was programmed as follows: 0–5 min, 5%B; 25 min, 30%B; 25.1–30 min, 80%B; 30.1–35 min, 5%B. The flow rate was set at 1 mL/min. Samples (10 *µ*L) were injected into the HPLC system and detected by using a diode array detector at the wavelength of 290 nm.

#### 2.9.3. Validation of the HPLC Method

Validation of the HPLC method was conducted according to the guideline of the International Conference on Harmonization (ICH 2005) [22]. The validation parameters included selectivity, linearity, accuracy, precision, limit of detection (LOD), and limit of quantitation (LOQ).

### 2.10. Statistical Analysis

The evaluations of anti-inflammatory activities were conducted in triplicate and the results were expressed as mean ± SEM. The IC_50_ values were calculated by using the Prism program. The data were analyzed using one-way ANOVA and Tukey's multiple comparison tests. The results of the HPLC analysis were expressed as mean ± SD. The level of statistical significance was taken at *P* < 0.05.

## 3. Results

### 3.1. Extraction of the Dried Leaves of *C. asiaticum*

Yields (%) of the ethanolic extracts of *C. asiaticum* leaves obtained from different preextraction treatments are shown in Table 1. The highest yield was obtained from the grilling method (CAG; 25.75%). Other methods, CAM, CAA, and CAC, yielded 24.23%, 22.88%, and 21.53%, respectively.

### 3.2. Anti-Inflammatory Activity by Inhibition of Nitric Oxide Production from RAW 264.7 Cells

As shown in Table 2, CAA showed the highest inhibitory activity with IC_50_ value of 16.66 ± 1.42 *µ*g/mL. CAM, CAG, and CAC exerted anti-inflammatory activity with IC_50_ values of 20.42 ± 0.57, 21.35 ± 0.56, and 22.48 ± 1.09 *µ*g/mL, respectively. All of them exhibited anti-inflammatory activity significantly less than the positive control, prednisolone (IC_50_ = 1.05 ± 0.13 *μ*g/mL). Lycorine, a major component of *C. asiaticum*, exerted potent activity with IC_50_ value of 0.40 ± 0.01 *μ*g/mL. On the contrary, diclofenac was not active in this pathway. The results demonstrated that CAA could affect significantly lower levels of NO production compared with CAG and CAC (Table 1).

### 3.3. Anti-Inflammatory Activity by Inhibition of TNF-*α* Production from RAW 264.7 Cells

The results in Table 1 showed that CAA exerted the highest activity with IC_50_ value of 25.21 ± 1.11 *µ*g/mL. CAM, CAG, and CAC showed anti-inflammatory activity with IC_50_ values of 37.60 ± 0.58, 46.36 ± 0.48, and 45.63 ± 1.54 *µ*g/mL, respectively. However, all of them have significantly less (*p* < 0.05) anti-inflammatory activity than prednisolone (IC_50_ = 0.07 ± 0.00 *μ*g/mL). Lycorine showed inhibition of 14.0% at the concentration of 0.8 *μ*g/mL. Higher lycorine concentrations showed >30% cytotoxicity to RAW 264.7 cell. On the contrary, diclofenac was not active in this pathway. The results showed that CAA could significantly lower the level of TNF-*α* production when compared with CAM, CAG, and CAC (*p* < 0.05).

### 3.4. Anti-Inflammatory Activity by Inhibition of PGE2 Production from RAW 264.7 Cells

As shown in Table 1, all extracts of *C. asiaticum* were not active in this pathway. However, prednisolone, the positive control, showed strong anti-inflammatory activity with IC_50_ value of 0.07 ± 0.00 *μ*g/mL. All extracts were significantly different from prednisolone (*p* < 0.05) in this respect. Diclofenac showed potent inhibition with IC_50_ value of 0.004 ± 0.001 *μ*g/mL while lycorine was not active in this pathway.

### 3.5. Cytotoxicity of Extracts on RAW 264.7 Cells by MTT Assay

Cytotoxicity on RAW 264.7 cells of the ethanolic extracts from different preextraction treatments of *C. asiaticum* leaf was determined by MTT assay. As shown in Table 2, all extracts were not toxic at all concentrations. Lycorine showed cytotoxicity at a concentration greater than 1 *μ*g/mL.

### 3.6. Total Phenolic Content

As shown in Table 1, CAA had the highest total phenolic content with the value of 23.20 ± 1.36 mg GAE/g. CAM, CAG, and CAC showed total phenolic content of 14.53 ± 0.63, 13.10 ± 1.02, and 12.01 ± 0.84 mg GAE/g, respectively.

### 3.7. High-Performance Liquid Chromatography (HPLC) Analysis and Method Validation

**Figure 2:**
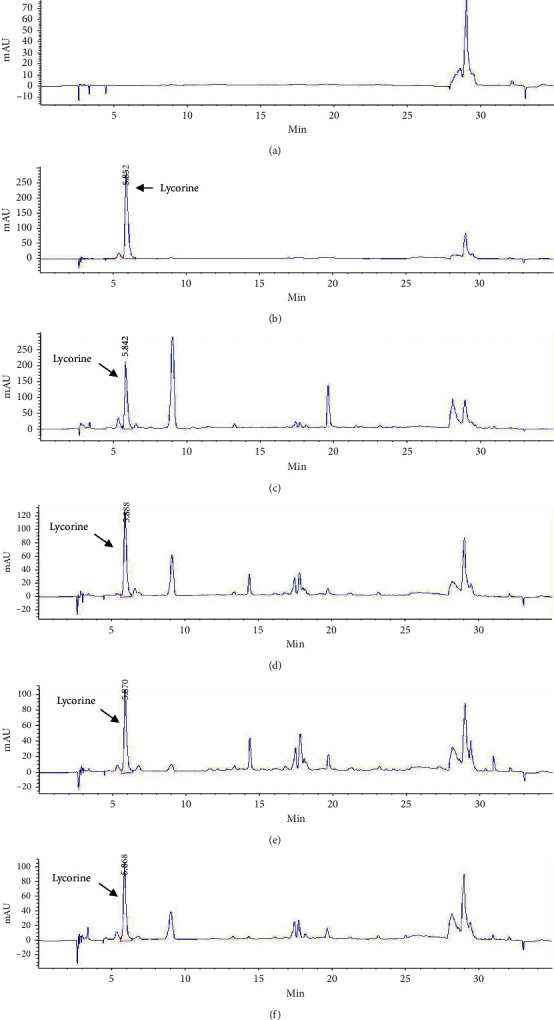
HPLC chromatograms of blank (DMSO) (a), standard lycorine solution (0.5 mg/mL) (b), CAA extract at 10 mg/mL (c), CAM extract at 10 mg/mL (d), CAG extract at 10 mg/mL, (e) and CAC extract at 10 mg/mL (f) by using HPLC analysis with mobile phase consisting of 0.1%v/v triethylamine in water adjusting pH to 3.0 with phosphoric acid (A) and acetonitrile (B). Gradient elution was programmed as follows: 0–5 min, 5%B; 25 min, 30%B; 25.1–30 min, 80%B; 30.1–35 min, 5%B and detected at the wavelength of 290 nm. The chromatograms of standard lycorine solution (b) and crude extracts solutions (c), (d), (e), and (f) show similar peaks at RT = 5.8.

**Figure 3:**
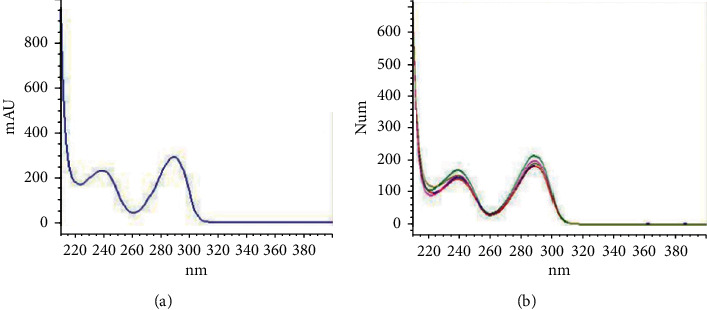
Comparing the UV absorption spectra of peak start, peak apex, and peak end at RT = 5.8 of lycorine standard (a), overlaying UV spectrum of each sample solution (CAA, CAM, CAG, and CAC) (b) by using HPLC analysis.

The HPLC system showed good separation of lycorine from other substances in the extract, the peak of lycorine matched that of the standard peak at RT = 5.8, and the blank (DMSO) showed no peak at the same RT in the standard (Figure 2). The chromatograms of the crude extracts and standard solutions paralleled those of the UV spectrum of the lycorine peaks. Peak purity of lycorine in the sample chromatograms was assessed by comparing the UV spectra at the start, apex, and end of the peak of lycorine standard. The results showed that the UV spectrum of each sample (CAA, CAM, CAG, and CAC) was similar to that of the lycorine standard (Figure 3).

**Figure 4:**
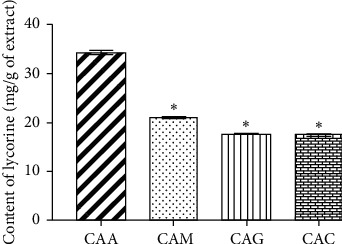
Comparison of lycorine contents (mg/g of extract) of the ethanolic extracts of *C. asiaticum* leaves after different preextraction treatments by using HPLC analysis. ^*∗*^Significant difference (*p* < 0.05) compared with CAA analyzed by Tukey's multiple comparisons test.

The HPLC system for lycorine analysis showed excellent linearity via the coefficient of determination (*r*^2^ = 1) within the range of 50–500 *μ*g/mL (Table 3). LOD and LOQ of lycorine were 1 and 3.25 *μ*g/mL, respectively (Table 3). The accuracy of the method was presented as %recovery intraday and interday within 99.76–101.96% and 99.07–100.62%, respectively (Table 4). The precision RSD% values were lower than 2%, exhibiting a great precision of the method (Table 4). The good linearity, accuracy, and precision of the HPLC method signify its suitability for the determination of lycorine in the ethanolic extracts of *C. asiaticum* leaves after different preextraction treatments. The comparative values of lycorine content of *C. asiaticum* leaf extracts after different preextraction treatments are shown in Figure 4. CAA, CAM, CAG, and CAC show the contents to be 34.40 ± 0.41, 21.05 ± 0.28, 17.71 ± 0.13, and 17.48 ± 0.24 mg/g, respectively.

## 4. Discussion

Osteoarthritis is a common ailment among the elderly in Thailand and elsewhere. The overproduction of NO, TNF-*α*, and PGE_2_ are found in inflamed articular tissues and human chondrocytes in OA patients [23, 24]. In addition, oxidation can lead to tissue injury, cartilage, and joints degeneration [25].

*C. asiaticum,* a medicinal plant in Phra-aung-kob-phra-sean remedy, has long been used to treat injuries and joint inflammation. Thai traditional/folk medicine uses grilled leaves of the herb for this purpose. The guidelines for certification of folk medicine describe that fresh leaves are grilled lightly by fire from a charcoal stove then pressed and wrapped around the pain area [26].

Previous phytochemical studies on *C. asiaticum* reported that alkaloids, coumarins, glycosides, triterpenes, phenolics, and flavonoids are constituents of *C. asiaticum* leaf [27, 28]. Lycorine is one of the major *Crinum* alkaloids [14, 28]. A previous study reported that ethanolic extract of the leaves exerted *in vitro* anti-inflammatory activity by the inhibition of nitric oxide production [11]. Leaf extract also showed a significant anti-inflammatory effect *in vivo* [12, 29]. Another study reported that methanol and chloroform extracts of hot air-dried leaf of *C. asiaticum* exerted antinociceptive effect [27]. To date, there has been no report on the effects of different preextraction treatments on its chemical properties and anti-inflammatory activity. Our research addressed this gap of knowledge by comparing the effects of four preextraction treatments of the leaf on its anti-inflammatory activity and chemical properties.

All extracts prepared by different preextraction treatments showed inhibition of NO production. The extract obtained from hot air oven-dried leaves (CAA) exerted the highest activity with IC_50_ value of 16.66 ± 1.42 *µ*g/mL. This compares well with the result from a previous study showing IC_50_ value of 9.99 ± 0.00 *µ*g/mL [11]. All extracts showed less inhibitory activity than the positive control, prednisolone. Extract from CAA also exerted the highest activity on TNF-*α* production with IC_50_ value of 25.21 ± 1.11 *µ*g/mL. Extracts from other preextraction treatments exerted less activity with IC_50_ higher than 35 *µ*g/mL. All extracts exerted less inhibition of TNF-*α* production than prednisolone. Diclofenac was not active in TNF-*α* pathway. None of the extracts inhibited PGE_2_ production. Our study showed that lycorine exerted strong anti-inflammatory activity by inhibiting NO production, but it only slightly inhibited TNF-*α* and PGE_2_ production in RAW264.7 cells. A previous study reported that lycorine from ethanolic extract of *Crinum yemense* inhibited LPS-induced iNOS but not COX-2 gene expression [30]. From another previous research [31], lycorine inhibited NO production in RAW 264 cells with IC_50_ value of 1.2 ± 0.4 *µ*M as compared with our result of 0.4 *µ*g/mL or 1.43 *µ*M. The difference in the results may be caused by the different assay procedures such as the concentrations of seeded cells in 96-well plates and the concentrations of LPS for inducing the nitric oxide production in RAW264.7 cells.

For the phytochemical study, we investigated the effect of various preextraction treatments of *C. asiaticum* leaf on total phenolic and lycorine contents. The CAA extract showed the highest total phenolic content (23.20 ± 1.36 mg GAE/g), as compared to the result in a previous research of 60.28 ± 0.38 TAE/g [10]. The difference may be due to factors such as season, temperature, and soil composition for plant growth as well as processing methods.

Phenolic compounds contribute to antioxidant activity [32, 33]. Oxidative stress activates inflammatory mediators linked to the overproduction of ROS involved in several chronic inflammatory diseases [34]. Therefore, phenolic compounds may contribute to the anti-inflammatory activity via their antioxidant effect. A recent study reported the antioxidant activity of ethanolic extracts of *C. asiaticum* leaf and bulk [35].

Lycorine content in ethanolic extracts of *C. asiaticum* leaves obtained from various preextraction treatments was determined using a validated HPLC method. The method showed good specificity, accuracy, and precision. CAA extract showed the highest lycorine content (34.40 ± 0.41 mg/g) which was significantly higher than that of CAM, CAG, and CAC extracts (*p* value < 0.05). Our results showed CAA extract exerted the highest inhibitory activity on NO production from LPS*-*induced RAW 264.7 cells. Lycorine has been shown to have a potent inhibitory activity on NO production. This suggested that lycorine may be the responsible marker of *C. asiaticum* leaves for inhibitory activity on NO production. NO, one of the inflammatory mediators important in the inflammation process which have a destructive effect on articular cartilage, helps bring about osteoarthritis which is the most common chronic degenerative disease. Additionally, lycorine has potent immunological, antitumor, and anti-inflammatory activities [14, 15] and has been shown to decrease autophagy in osteoclasts in vivo [16]. Therefore, lycorine is most likely the responsible active compound in *C. asiaticum* extract thus supporting the traditional use of *C. asiaticum* in the mitigation of joint pain.

Due to lycorine cytotoxicity to RAW264.7, its inhibitory activity on TNF-*α* and PGE_2_ production could not be demonstrated, though it exerted inhibition at a concentration higher than 0.8 *μ*g/mL. However, lycorine is not the only active compound in *C. asiaticum*; there are other compounds [36, 37] but they have not been investigated, which should be a subject for future research.

Results of this study confirmed our hypothesis that different preextraction treatments affected the anti-inflammatory activities and chemical properties of *C. asiaticum* leaves. The extract obtained after hot air (40°C) oven drying (CAA) showed the highest inhibitory activity on NO and TNF-*α* production, and total phenolic and lycorine contents. Extracts from other preextraction treatments were significantly lower, especially the traditional grilling on a charcoal stove (CAG) and temperature shock in hot and cold water before hot air drying (CAC). It appears that in the case of charcoal stove, factors such as high temperature, smoke, and ashes formed on the leaf surface may cause the degradation of the active compounds. Charcoal on the grilled leaf may adsorb the chemical constituents causing a decrease in total phenolic content and other bioactive components [38]. For the CAC method, the leaves were directly dipped into boiling water (approx. 100°C) and then ice water (approx. 0°C). This process may cause the degradation and leaching of some bioactive compounds. The extract from the CAC method showed the lowest lycorine content. The microwave drying method (CAM) is the technique that transforms electromagnetic energy into thermal energy. The electromagnetic energy may affect some compounds at the molecular level and the rapid heating of the material may cause thermal degradation of heat-sensitive components. A previous report showed that total phenolic content was decreased by microwave heating [39, 40]. Moreover, another study revealed that lycorine was unstable to light, oxygen, and heat. Since ring C of this alkaloid contains a double bond and at least one hydroxyl group, amino function and aromatization of this ring do occur under a variety of reaction conditions [41]. Hence, the results of our study suggested that different preextraction treatments affect the anti-inflammatory activity and chemical properties of *C. asiaticum* leaves differently, some of which are quite detrimental.

Results of this study support the use of *C. asiaticum* in Thai traditional medicine for pain relief in osteoarthritis. Hot air drying in an oven at 40°C for 10 hours should be recommended as a preextraction treatment for *C. asiaticum* leaves for its simplicity and best retention of the herbal quality. It can also be easily scaled up to industrial level for further development of *C. asiaticum* as a drug to treat joint pain in osteoarthritis. However, more study may be needed to investigate other bioactive compounds in the extract and the mechanisms through which they mitigate the pain.

## 5. Conclusion

Our study showed that air drying at 40°C is the best preextraction treatment for *C. asiaticum* leaves prior to 95% ethanolic extraction. It produced the extract with the highest anti-inflammatory activity as shown by its ability to inhibit nitric oxide and TNF-*α* production in RAW264.7 cells and was not toxic to the cells. The extract also had the highest total phenolic and lycorine contents which are antioxidants that influence its anti-inflammatory activity. Therefore, this study supports the traditional use of *C. asiaticum* to mitigate joint pain and hot air drying should be used as the preextraction treatment.

## Figures and Tables

**Table 1:** Yields (%w/w), anti‐inflammatory activity by the inhibitory effect on three pathways in RAW 264.7 cells, and %total phenolic content of the ethanolic extracts from different preextraction treatments of *C. asiaticum*.

Sample	%yields	Anti-inflammatory activity by inhibitory effect on three pathways in RAW 264.7 cells (IC_50_ ± SEM) (*μ*g/mL)			%Total phenolic content (mg GAE/g)
		NO	TNF-*α*	PGE_2_	
CAA	22.88	16.66 ± 1.42	25.21 ± 1.11	>100	23.20 ± 1.36
CAM	24.23	20.42 ± 0.57	37.60 ± 0.58^∗^	>100	14.53 ± 0.63^*∗*^
CAG	25.75	21.35 ± 0.56^∗^	46.36 ± 0.48^∗^	>100	13.10 ± 1.02^*∗*^
CAC	21.53	22.48 ± 1.09^∗^	45.63 ± 1.54^∗^	>100	12.01 ± 0.84^*∗*^
Prednisolone	—	1.05 ± 0.13	0.07 ± 0.00	0.07 ± 0.00	—
Diclofenac	—	>100	>100	0.004 ± 0.001	—
Lycorine	—	0.40 ± 0.00	>0.8	>0.8^(t)^	—

^∗^Significant difference (p < 0.05) compared with CAA analyzed by Tukey's multiple comparison test. _(t)_Cytotoxicity was observed at a higher concentration.

**Table 2:** Inhibitory effect of the ethanolic extracts from different preextraction treatments of *C. asiaticum* on LPS-induced NO production from RAW 264.7 cells (mean ± SEM, *n* = 3).

Sample	% inhibition of NO production from RAW 264.7 cells at various concentrations (*µ*g/mL) (% cytotoxicity)									IC50 ± SEM (µg/mL)
	0.1	0.2	0.4	0.8	1	10	30	50	100	
CAA	—	—	—	—	-2.58 ± 2.36	23.49 ± 5.96	96.48 ± 1.82	97.49 ± 1.23	98.26 ± 0.50	16.66 ± 1.42
					(−6.08 ± 12.47)	(−10.10 ± 9.18)	(2.95 ± 0.82)	(−15.27 ± 9.52)	(23.91 ± 0.85)	
CAM	—	—	—-	—	−10.80 ± 8.07	11.03 ± 2.75	83.44 ± 4.58	102.39 ± 5.33	96.12 ± 0.56	20.42 ± 0.57
					(−0.63 ± 8.29)	(−3.40 ± 2.59)	(8.07 ± 1.05)	(9.45 ± 0.52)	(12.00 ± 5.64)	
CAG	—	—	—	—	−4.63 ± 1.18	6.78 ± 2.44	83.37 ± 1.62	96.09 ± 0.37	101.55 ± 3.49	21.35 ± 0.56
					(−1.94 ± 6.54)	(2.62 ± 6.01)	(−5.60 ± 12.94	(3.72 ± 7.17)	(10.41 ± 8.68)	
CAC	—	—	—	—	−6.33 ± 1.28	−1.25 ± 7.92	81.21 ± 2.68	96.61 ± 2.71	96.59 ± 0.99	22.48 ± 1.09
					(2.33 ± 6.41)	(0.92 ± 2.94)	(7.23 ± 2.52)	(11.66 ± 5.08)	(17.07 ± 4.07)	
Prednisolone	32.85 ± 7.64	—	—	—	48.04 ± 2.07	64.11 ± 7.46	—	79.16 ± 3.18	—	1.05 ± 0.13
	(2.02 ± 3.15)				(5.35 ± 3.43)	(3.88 ± 4.68)		(9.64 ± 4.74)		
Diclofenac	—	—	—	—	—	—	—	−8.57 ± 0.86	1.16 ± 0.90	>100
								(−20.36 ± 0.68)	(11.88 ± 1.49)	
Lycorine	−10.93 ± 2.34	7.46 ± 0.44	50.72 ± 0.37	82.63 ± 3.90	86.86 ± 3.66^(t)^	—	—	—	—	0.40 ± 0.00
	(5.46 ± 4.37)	(9.98 ± 8.20)	(15.29 ± 4.92)	(27.35 ± 1.30)	(36.96 ± 4.15)					

(—) means not tested. ^(t)^cytotoxicity greater than 30%.

**Table 3:** Linearity, range, LOD, and LOQ of lycorine.

Validation parameters	Lycorine
Linear equation (*y* = ax + *b*)	*y* = 8.2971*x* + 46.55
Linearity (*r*^2^)	1
Range (*µ*g/mL)	50–500
LOD (*µ*g/mL)	1
LOQ (*µ*g/mL)	3.25

**Table 4:** Accuracy and precision validation of the analytical method for lycorine.

Lycorine	Spiked concentration (*μ*g/mL)	Measured concentration (*μ*g/mL; mean ± SD)	% RSD	% accuracy
Intraday (*n* = 3)	100	99.82 ± 1.20	1.21	99.82
	150	152.93 ± 1.83	1.19	101.96
	300	299.29 ± 1.38	0.46	99.76
Interday (*n* = 9)	100	99.07 ± 1.03	0.89	99.07
	150	150.93 ± 2.42	1.44	100.62
	300	300.08 ± 1.16	0.38	100.03

## Data Availability

The data used to support the findings of this study are included within the article.
